# Mechanical overloading causes mitochondrial superoxide and SOD2 imbalance in chondrocytes resulting in cartilage degeneration

**DOI:** 10.1038/srep11722

**Published:** 2015-06-25

**Authors:** Masato Koike, Hidetoshi Nojiri, Yusuke Ozawa, Kenji Watanabe, Yuta Muramatsu, Haruka Kaneko, Daichi Morikawa, Keiji Kobayashi, Yoshitomo Saita, Takahisa Sasho, Takuji Shirasawa, Koutaro Yokote, Kazuo Kaneko, Takahiko Shimizu

**Affiliations:** 1 Department of Advanced Aging Medicine, Chiba University Graduate School of Medicine, Chiba, Japan; 2 Department of Orthopaedics, Juntendo University Graduate School of Medicine, Tokyo, Japan; 3 Department of Orthopaedics, Chiba University Graduate School of Medicine, Chiba, Japan; 4 Department of Aging Control Medicine, Juntendo University Graduate School of Medicine, Tokyo, Japan; 5 Department of Clinical Cell Biology and Medicine‚ Chiba University Graduate School of Medicine, Chiba, Japan

## Abstract

Mechanical stress and aging are major risk factors of cartilage degeneration. Human studies have previously reported that oxidative damage increased, while SOD2 protein was reciprocally downregulated in osteoarthritic degenerated cartilage. However, it remains unclear whether mitochondrial superoxide imbalance in chondrocytes causes cartilage degeneration. We herein demonstrate that mechanical loading promoted mitochondrial superoxide generation and selective *Sod2* downregulation in chondrocytes *in vivo* and that mitochondrial superoxide inducer also downregulated *Sod2* expression in chondrocytes *in vitro*. A genetically manipulated model revealed that *Sod2* deficiency in chondrocytes also resulted in mitochondrial superoxide overproduction and dysfunction, thus leading to cartilage degeneration. Intra-articular injection of a permeable antioxidant effectively suppressed the mechanical loading-induced mitochondrial superoxide generation and cartilage degeneration in mice. Our findings demonstrate that mitochondrial superoxide plays a pivotal role in the development and progression of osteoarthritis, and the mitochondrial superoxide balance may therefore be a promising target for the treatment of cartilage degeneration.

Osteoarthritis (OA) is the most common joint disease in the United States^1^, which is mainly characterized by cartilage degeneration. The research on osteoarthritis against disability (ROAD) study in 2009, a Japanese large-scale population-based cohort study, showed that the prevalence of knee OA in men and women was 42.0% and 61.5%, respectively, in patients older than 40 years of age, and the estimated number of knee OA patients was approximately 25 million in Japan^2^. Articular cartilage consists of a large matrix with a chondrocyte, which has a layered structure divided into four zones: the superficial zone, the middle zone, the deep zone, and the zone of calcified cartilage. Type II collagen and aggrecan are major components of the extracellular matrix (ECM) in articular cartilages. Hyaluronic acid therapy is frequently used to treat knee osteoarthritis (OA) via an intra-articular injection. Although several studies have reported the therapeutic effect of the intra-articular injection of hyaluronic acid^3^,^4^,^5^, the beneficial effect of this therapy in knee OA development was limited^3^. Basic scientific studies have assumed that matrix-degrading enzymes such as matrix metalloproteinase-3 (MMP3), matrix metalloproteinase-13 (MMP13), and a disintegrin and metalloproteinase with thrombospondin motifs-5 (ADAMTS5), are important factors for OA development^6^,^7^,^8^. However, pharmacological benefits for OA development using MMP and ADAMTS5 inhibitors in humans were not observed^9^. Thus, there is currently no useful therapy to treat cartilage degeneration.

Mechanical stress and aging are major risk factors for OA development^10^,^11^. Growing evidence has suggested that excessive loading induced by a malaligned knee joint and being overweight led to cartilage degeneration^12^,^13^,^14^,^15^. Interestingly, Wolff *et al.* reported that cyclic dynamic loading induced superoxide generation in the chondrocytes of bovine osteochondral explants using a compression apparatus *in vitro*^16^, suggesting that superoxide plays a pathological role in mechanical loading-induced OA development. Several reports have also found that an oxidative damage marker, nitrotyrosine, which is a byproduct of peroxynitrite formed from superoxide and nitric oxide, was significantly increased in human OA cartilage^17^,^18^,^19^, thus supporting the pathological significance of oxidative damage in OA.

Superoxide is a reactive oxygen species (ROS), which are harmful molecules mainly generated during mitochondrial respiration and metabolized by superoxide dismutase 2 (SOD2) in the mitochondria. Biochemical analyses using degenerative cartilage from OA patients have suggested a pathological relationship between SOD2 downregulation and cartilage degeneration in OA progression, suggesting that the *Sod2*-centric redox balance in the mitochondria was associated with OA^20^,^21^,^22^. However, the protective role of SOD2 and the pathological role of mitochondrial superoxide in OA have not yet been fully elucidated. We aim to elucidate whether abnormal loading causes a mitochondrial imbalance by *Sod2* loss in chondrocytes and whether the loss of *Sod2* or mitochondrial superoxide overproduction accelerates cartilage degeneration in mice.

## Results

### Mechanical overloading elevated mitochondrial superoxide generation and downregulated *Sod2* expression in knee chondrocytes

**Figure 1:**
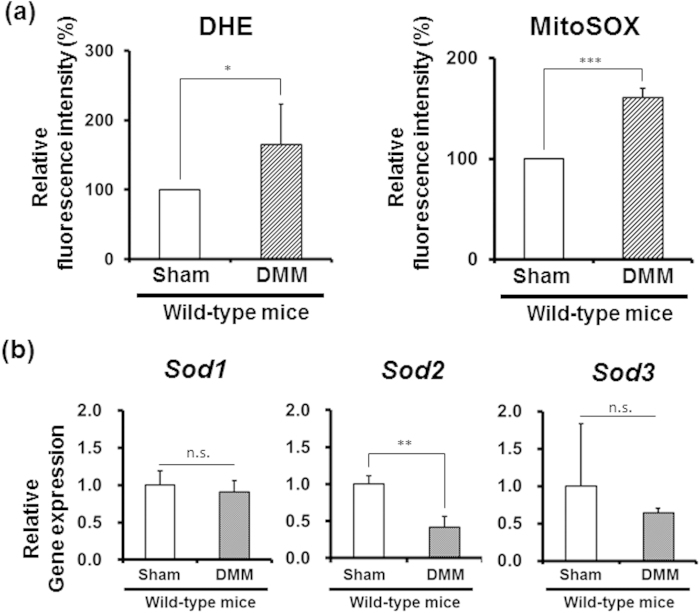
Abnormal loading induces mitochondrial superoxide level and selective downregulation of *Sod2* in chondrocytes *in vivo*. (**a**) Flow cytometric analyses of superoxide generation in wild-type chondrocytes isolated from tibial plateau at 2 weeks after DMM surgery. Left and right panels indicate findings of DHE and MitoSOX staining, respectively. Sham: sham-surgery, DMM: DMM surgery (*n* = 5, *P < 0.05, ***P < 0.001 versus sham, Student’s *t*-test). Error bars show the mean ± s.d. (**b**) Gene expression of superoxide-degrading enzymes in wild-type chondrocytes isolated from the tibial plateau at 2 weeks after DMM surgery. Sham: sham-surgery, DMM: DMM surgery. Error bars show the mean ± s.d. (*n* = 3, **P < 0.01 versus sham, n.s.: not significant, Student’s *t*-test).

The surgical destabilization of the medial meniscus (DMM) was created to induce abnormal loading in the chondrocytes of knee cartilage of C57BL/6 wild-type mice at 20 weeks of age as previously described^23^. The cartilage sections showed early focal degeneration at 2 weeks after DMM surgery^24^,^25^. To reveal whether superoxide in the chondrocytes is increased under mechanical loading before obvious cartilage degeneration, superoxide generation in articular chondrocytes from both the sham surgery and DMM sides was evaluated at 2 weeks after surgery using dihydroethidium (DHE) and MitoSOX staining, which are detectors of intracellular and mitochondrial superoxide, respectively. Flow cytometric analysis revealed that the instability treatment significantly induced intracellular and mitochondrial superoxide generation in chondrocytes from the DMM side (Fig. 1a).

Furthermore, we evaluated the gene expression of several superoxide-degrading enzyme genes, including *Sod1, Sod2,* and *Sod3,* to clarify the reducing capacity in chondrocytes in an instability murine model. Interestingly, DMM treatment selectively decreased *Sod2* expression in wild-type chondrocytes (Fig. 1b). These results indicated that the mechanical loading enhanced the cellular and mitochondrial superoxide levels associated with *Sod2* downregulation, leading to a mitochondrial superoxide imbalance in chondrocytes *in vivo*.

### Mitochondrial superoxide overproduction by paraquat treatment impaired *Sod2* expression and mitochondrial function in chondrocytes

**Figure 2:**
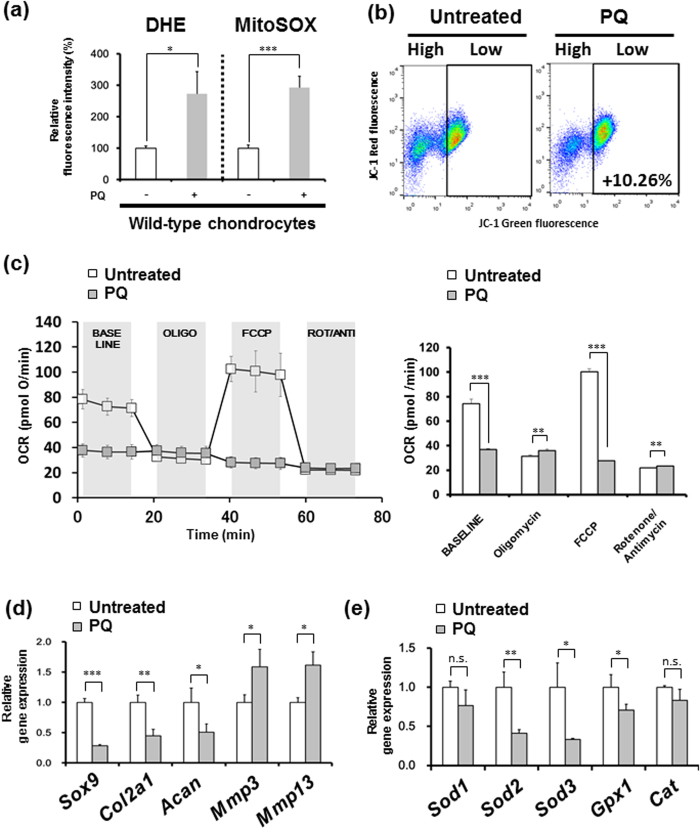
Mitochondrial superoxide deregulation by paraquat causes *Sod2* downregulation and mitochondrial dysfunction in chondrocytes. (**a**) Superoxide generation in primary wild-type chondrocytes at culture day 6 after 1 mM PQ treatment for 24 h (*n* = 3, *P < 0.05, ***P < 0.001 versus control, Student’s *t*-test). (**b**) Mitochondrial membrane potential (ΔΨm) in primary wild-type chondrocytes at culture day 6 after 1 mM PQ treatment for 24 h. High: the region of cells with normal ΔΨm, Low: the region of cells with mitochondrial depolarization. (**c**) OCR in primary wild-type chondrocytes at culture day 6 after 1 mM PQ treatment for 24 h. Respirometry shows the sequential addition of mitochondrial metabolic inhibitors. BASELINE: the basal OCR, OLIGO: the OCR after inhibition of ATP synthase with the addition of oligomycin (final concentration: 1 μM), FCCP: the maximal stimulatory capacity after the addition of FCCP (final concentration: 0.5 μM) treatment, ROT/ANTI: the OCR after the addition of rotenone/antimycin (final concentration: 1 μM each) treatment. The right graph indicates the quantification of the OCR following the sequential addition of mitochondrial metabolic inhibitors (*n* = 3, respectively, **P < 0.01 versus control, Student’s *t*-test). (**d**, **e**) Gene expression profiles of antioxidant enzymes (**d**) and OA-related genes (**e**) in primary wild-type chondrocytes at culture day 6 after 1 mM PQ treatment for 24 h (each *n* = 3, *P < 0.05, **P < 0.01, ***P < 0.001 versus control, n.s.: not significant, Student’s *t*-test).

Paraquat (PQ, methyl viologen dichloride hydrate) is well known as a mitochondrial superoxide inducer at complex I^26^. To confirm whether mitochondrial superoxide impairs the chondrocyte function directly, primary wild-type chondrocytes were treated with 1 mM PQ for 24 h. Initially, we evaluated whether PQ induces mitochondrial superoxide in chondrocytes. The superoxide production in chondrocytes was measured using flow cytometry with DHE and MitoSOX stainings. PQ treatment significantly induced superoxide generation in the mitochondria of chondrocytes (Fig. 2a). To evaluate whether PQ induces mitochondrial depolarization and impairs mitochondrial respiration in chondrocytes, we next analyzed the mitochondrial membrane potential (ΔΨm) in chondrocytes using flow cytometry with JC-1 staining. After 1 mM PQ treatment for 24 h, PQ increased the mitochondrial depolarization in wild-type chondrocytes (Fig. 2b). Furthermore, an extracellular flux analysis showed that the oxygen consumption rate (OCR) in wild-type chondrocytes was significantly decreased by the PQ treatment (Fig. 2c). These results demonstrated that mitochondrial superoxide induced mitochondrial dysfunction.

Furthermore, to evaluate whether PQ impairs the ECM homeostasis in chondrocytes, we investigated the gene expression of OA-related genes. As expected, anabolic genes (including *Sox9, Col2a1,* and *Acan*) were significantly downregulated, while catabolic genes (including *Mmp3* and *Mmp13*) were significantly upregulated (Fig. 2d), thus indicating an ECM imbalance. To investigate whether superoxide affects the expression pattern of antioxidant enzymes, the gene expression of the major antioxidant enzymes in articular chondrocytes were evaluated after 1 mM PQ treatment for 24 h. Interestingly, *Sod2, Sod3,* and *Gpx1* were significantly decreased, while the expression of *Sod1* and *Cat* did not change in PQ-treated chondrocytes (Fig. 2e).

Taken together, these results indicated that the mitochondrial superoxide overproduction following PQ treatment resulted in mitochondrial dysfunction and suppressed the gene expression of antioxidant enzymes, including *Sod2*, leading to an impaired ECM metabolism.

### Depletion of *Sod2* in chondrocytes spontaneously accelerated cartilage degeneration during aging

**Figure 3:**
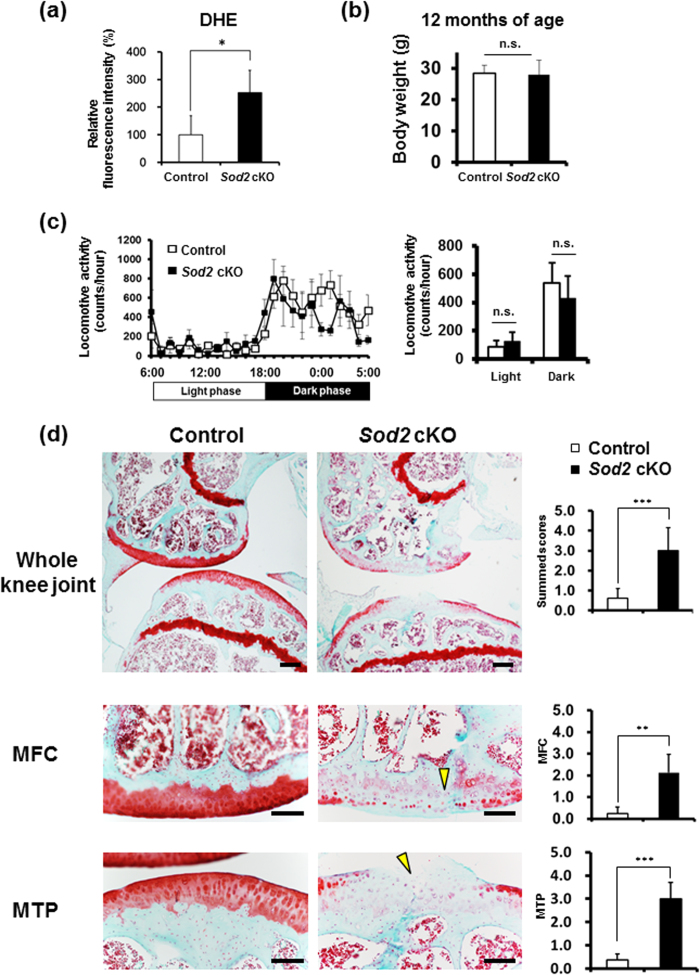
Loss of *Sod2* in chondrocytes exacerbates cartilage degeneration during aging. (**a**) Quantification of superoxide generation in tibial articular chondrocytes of *Sod2* cKO mice at 10 weeks of age using flow cytometry with DHE staining. Error bars show the mean ± s.d. (*n* = 5, *P < 0.05 versus sham, Student’s *t*-test). (**b**) Body weight of control and *Sod2* cKO male mice at 12 month of age. Values are the mean ± s.d. (*n* = 6, n.s., not significant versus control mice, Student’s *t*-test). (**c**) Locomotive activity of control and *Sod2* cKO male mice at 12 months of age. The right graph indicates the quantification of locomotive activity in control littermates (*Sod2*^*fl/fl*^) and *Sod2* cKO mice. Values are the mean ± s.e.m. (*n* = 6, n.s., not significant versus control mice, Student’s *t*-test). (**d**) Cartilage degeneration in safranin O/Fast Green stained sections of the medial region of knee joints from control littermates and *Sod2* cKO mice at 12 months of age. Yellow arrowheads indicate the vertical cleft region. The right upper graph indicates the quantification of cartilage degeneration in the medial region, including the medial femoral condyle (MFC) + medial tibial plateau (MTP) of the knee joint; the right middle graph indicates the quantification of cartilage degeneration in MFC; and the right lower graph indicates the quantification of cartilage degeneration in MTP. Scale bars represent, 1 mm. Values are the mean ± s.d. of four mice per group (**P < 0.01, ***P < 0.001 versus control, Student’s *t*-test).

We generated chondrocyte-specific *Sod2*-deficient mice (*Col2a1-Cre;Sod2*^*fl/fl*^, *Sod2* cKO) using a cre-loxP system to investigate the OA phenotypes of cartilage and chondrocytes in mice (Supplementary Fig. S1a). *Sod2* cKO mice were born at the expected Mendelian ratio and showed no obvious skeletal abnormalities at the developmental and growth phases (Supplementary Fig. S1a, b). Furthermore, the western blot analysis revealed a specific loss of SOD2 protein in the cartilage on postnatal day 6 *in vivo* (Supplementary Fig. S1c). The *Sod2* deficiency in chondrocytes significantly increased the superoxide level compared with that of control mice (Fig. 3a). However, the protein levels of another enzyme were not changed in *Sod2* cKO chondrocytes *in vitro* (Supplementary Fig. S2), indicating that *Sod2* loss did not induce the compensatory expression of other antioxidant enzymes.

To evaluate whether *Sod2* loss in chondrocytes accelerates cartilage degeneration during aging, we histologically analyzed the knee joints of *Sod2* cKO and control littermates (*Sod2*^*fl/fl*^ mice) at 12 months of age. Because cartilage degeneration is affected by body weight and locomotive activity, we first compared the differences in body weight and locomotive activity between *Sod2* cKO and control mice at 12 months of age. There were no significant differences seen in the body weight or spontaneous locomotive activity between *Sod2* cKO and control littermates at 12 months of age (Fig. 3b,c), indicating that *Sod2* cKO mice had normal growth and physical activity until 12 months of age. Histological analyses by the OARSI score revealed that *Sod2* cKO joints exhibited a significant loss of safranin-O staining in all layers of both articular cartilages in *Sod2* cKO mice (right panels of Fig. 3d). Surprisingly, we often observed vertical clefts/erosion in the calcified cartilage of the medial femoral condyle (MFC) and medial tibial plateau (MTP). In contrast, proteoglycan was present in all layers of the articular cartilage in control joints (left panels of Fig. 3d). These findings demonstrated that *Sod2* loss spontaneously accelerated cartilage degeneration and injury in knee joints during aging.

### Ablation of *Sod2* in chondrocytes promoted cartilage degeneration under mechanical loading

**Figure 4:**
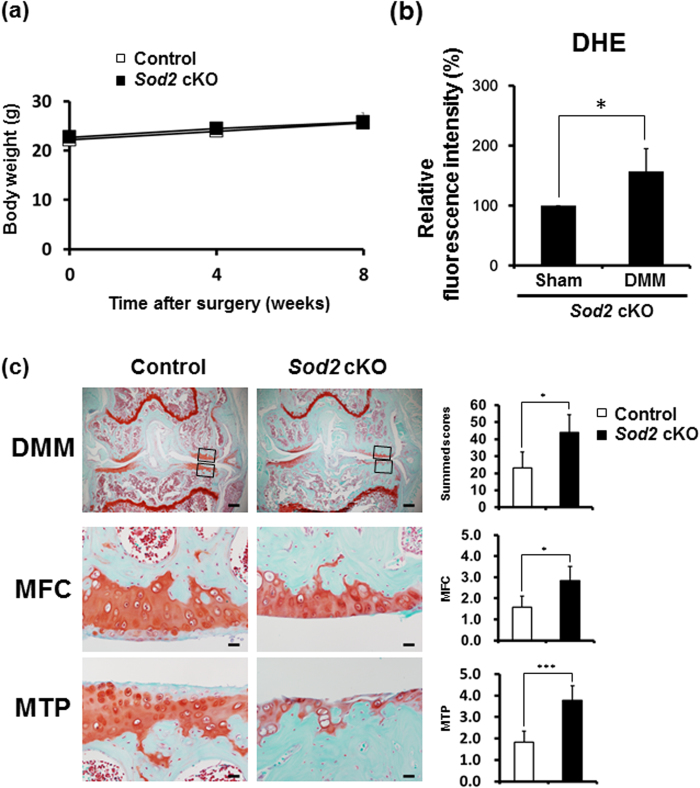
Loss of *Sod2* in chondrocytes causes cartilage degeneration under an instability condition. (**a**) Change in body weight of control and *Sod2* cKO mice after DMM surgery (*n* = 7 for each time point). (**b**) Quantification of superoxide generation in tibial articular chondrocytes at two weeks after DMM surgery using flow cytometry with DHE staining. Error bars show the mean ± s.d. (*n* = 5, *P < 0.05 versus sham, Student’s *t*-test). (**c**) Cartilage degeneration in safranin O/Fast Green stained sections of the knee joint from control littermates and *Sod2* cKO mice at eight weeks after DMM surgery. The right upper graph indicates the quantification of cartilage degeneration in whole knee joints; the right middle graph indicates the quantification of cartilage degeneration in MFC; and the right lower graph indicates the quantification of cartilage degeneration in MTP. Scale bars represent 200 μm and 20 μm for the low (top panels) and high (middle and low panels) magnifications, respectively. Values are the mean ± s.d. of five mice per group (*P < 0.05, ***P < 0.001 versus control, Student’s *t*-test).

To conclude whether chondrocytes without SOD2 enzyme are vulnerable to mechanical loading, DMM was created in the knee joints of *Sod2* cKO and control littermates at 8 weeks of age. After surgery, no significant differences were seen in the body weight between *Sod2* cKO and control littermates (Fig. 4a), excluding the adverse effects caused by surgery. Two weeks after the DMM surgery, we also measured the superoxide generation in chondrocytes isolated from the adult mutant cartilage. DMM treatment additionally enhanced the superoxide level in the DMM side of *Sod2* cKO cartilage (Fig. 4b). Eight weeks after the DMM surgery, we histologically evaluated by the OARSI score on knee joints sections. The control joint showed the loss of safranin-O staining without morphological changes in the superficial layer of MFC and MTP (left panels of Fig. 4c). Conversely, *Sod2* cKO joints exhibited markedly extended cartilage loss in the tide mark (right panels of Fig. 4c). These results demonstrated that the loss of SOD2 under an instability stress additively promoted mitochondrial superoxide production and exacerbated cartilage degeneration, thereby comparable to human OA pathologies in *Sod2* cKO cartilage.

### Mitochondrial superoxide produced by *Sod2* loss impaired extracellular matrix homeostasis via mitochondrial dysfunction

**Figure 5:**
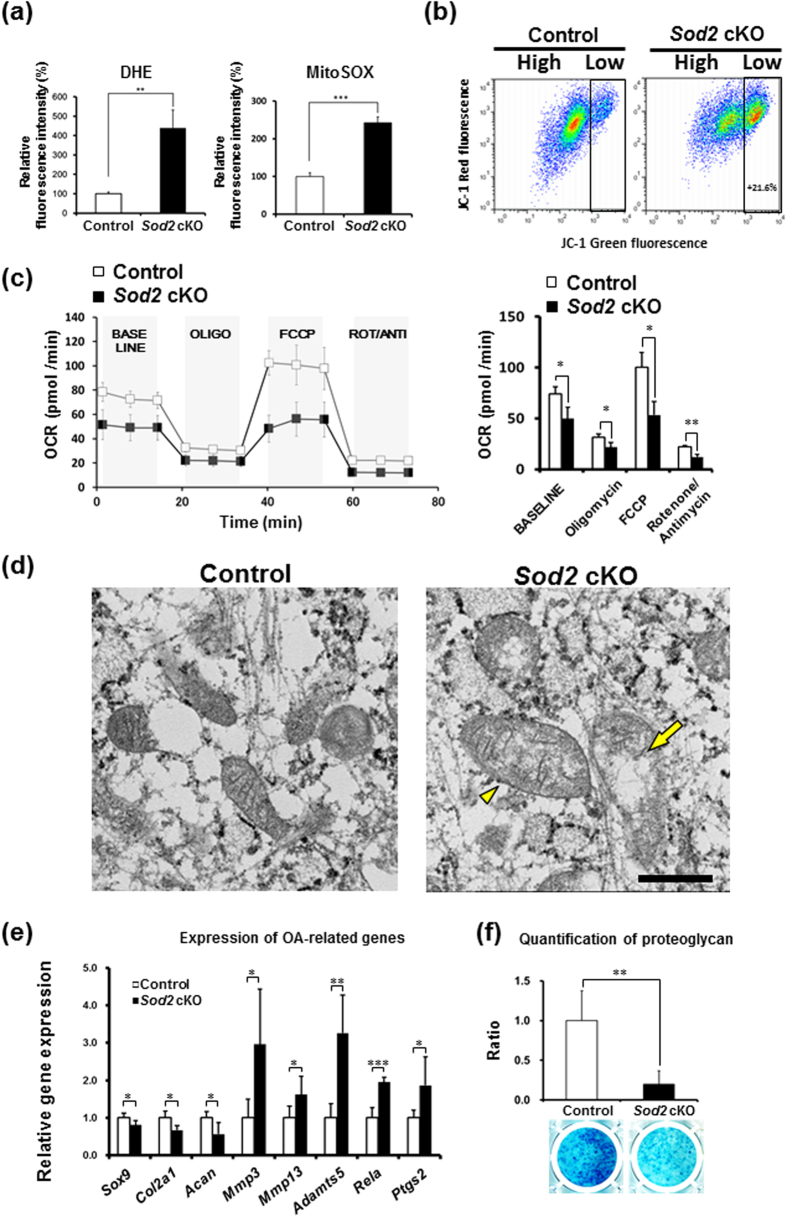
*Sod2* deficiency in chondrocytes induces mitochondrial superoxide generation and dysfunction resulting in impaired extracellular matrix metabolism. (**a**) Quantification of mitochondrial superoxide generation in *Sod2* cKO chondrocytes at culture day 6. The left and right panels indicate DHE and MitoSOX staining, respectively. Values are the mean ± s.d. of five per each group (**P < 0.01, ***P < 0.001 versus control, Student’s *t*-test). (**b**) Mitochondrial membrane potential (ΔΨm) in primary chondrocytes at culture day 6 with JC-1 staining. High: the region of cells with normal ΔΨm, Low: the region of cells with mitochondrial depolarization. The proportion of cells with low ΔΨm in *Sod2* cKO chondrocytes significantly increased at an average 21.6% compared with the controls. (**c**) OCR in primary *Sod2* cKO chondrocytes. High: the region of cells with normal ΔΨm, Low: the region of cells with mitochondrial depolarization. Respirometry shows the sequential addition of mitochondrial metabolic inhibitors as described in Fig. 2 legend. The right graph indicates the quantification of the OCR following the sequential addition of mitochondrial metabolic inhibitors (*n* = 3, respectively, *P < 0.05, **P < 0.01 versus control, Student’s *t*-test). (**d**) Mitochondrial morphology in *Sod2* cKO (right) and control (left) chondrocytes using an electron microscope. Scale bar represents 500 nm. A yellow arrowhead indicates a swollen mitochondria, a yellow arrow indicates disrupted mitochondrial cristae. (**e**) Expression profiles of OA-related genes in primary articular chondrocytes at culture day 6. Values are the mean ± s.d. of five per group (*P < 0.05, **P < 0.01, ***P < 0.001 versus control, Student’s *t*-test). (**f**) Proteoglycan levels in *Sod2* cKO chondrocytes at culture day 28 using Alcian blue staining. Quantification of Alcian blue staining was achieved by QWin software (*n* = 3, **P < 0.01 versus control, Student’s *t*-test).

In order to clarify the biological consequence of *Sod2* deficiency in chondrocytes *in vitro*, cellular phenotypes were evaluated in primary articular chondrocytes isolated from the knee joint of neonates. At culture day 6, superoxide generation, mitochondrial function, and the expression profiles of OA-related genes were evaluated. At day 28 of culture, the proteoglycan levels were quantified with Alcian blue staining. Initially, *Sod2* cKO chondrocytes demonstrated significantly increased superoxide generation via flow cytometry with DHE and MitoSOX stainings (Fig. 5a). The *Sod2* deficiency in chondrocytes also led to a higher proportion of cells with mitochondrial depolarization as evidenced through with JC-1 staining (Fig. 5b). Additionally, *Sod2* cKO chondrocytes showed that OCR was significantly decreased compared with the control chondrocytes using an extracellular flux analyzer (Fig. 5c). Furthermore, an electron microscopic analysis revealed swollen mitochondria with disrupted cristae in the *Sod2* cKO chondrocytes (right panel of Fig. 5d). These results indicated that mitochondrial superoxide overproduction impaired the mitochondrial function and morphology.

To determine whether mitochondrial superoxide accumulation impairs ECM metabolism, we evaluated the gene expression profiles of OA-related genes in *Sod2* cKO chondrocytes. Anabolic genes (including *Sox9, Col2a1,* and *Acan*) were significantly downregulated, while catabolic genes (including *Mmp3, Mmp13,* and *Adamts5*) were significantly upregulated (Fig. 5e). Furthermore, inflammation-related genes, including *Rela* and *Ptgs2*, were significantly upregulated (Fig. 5e). Furthermore, Alcian blue staining revealed a significant decrease of proteoglycan in the *Sod2* cKO chondrocytes at day 28 of culture (Fig. 5f). These results indicated that superoxide accumulation in the mitochondria impaired ECM metabolism via the deregulating gene expression of matrix-related genes. We also confirmed similar cellular phenotypes in tamoxifen-inducible *Sod2*-deficient chondrocytes from *Rosa26-Cre*^*ERT2*^*;Sod2*^*fl/fl*^ mice (Supplementary Figs S3 and S4).

### A vitamin C-permeable derivative suppressed mitochondrial superoxide generation and ameliorated cartilage degeneration *in vivo*

**Figure 6:**
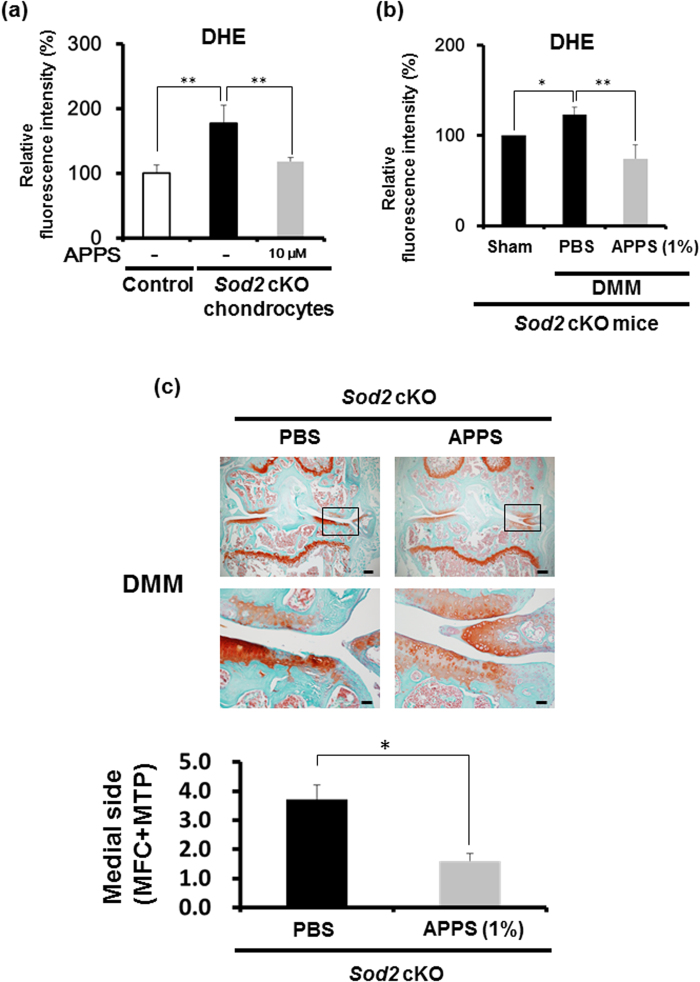
Antioxidant treatment effectively suppresses excessive mitochondrial superoxide generation and improves cartilage degeneration in *Sod2* cKO articular chondrocytes. (**a**) Superoxide generation in primary *Sod2* cKO chondrocytes at culture day 6 after 10 μM vitamin C derivative (APPS) treatment for 24 h (*n* = 4, **P < 0.01 versus *Sod2* cKO chondrocytes, Student’s *t*-test). (**b**) Superoxide generation in chondrocytes isolated from the tibial plateau of *Sod2* cKO cartilage after the intra-articular injection of 1% APPS or PBS for 24 h was measured via flow cytometry. APPS or PBS were injected into the joint cavity on the DMM side in *Sod2* cKO mice at 2 weeks after DMM surgery (*n* = 5, **P < 0.01 versus control, Student’s *t*-test). (**c**) Cartilage degeneration in safranin O/Fast Green stained sections of the knee joint from control littermates and *Sod2* cKO mice at eight weeks after DMM surgery following weekly intra-articular injections of 1% APPS or PBS. The bottom graph indicates the quantification of cartilage degeneration in the medial region of the knee joint in *Sod2* cKO mice. Scale bars represent 200 μm and 50 μm for the low (top panels) and high (low panels) magnifications, respectively (*n* = 5–7, *P < 0.05 versus control, Student’s *t*-test).

Vitamin C is known as a strong antioxidant without adverse effects and is necessary for maintaining cartilage to promote collagen biosynthesis^27^. However, vitamin C is easily oxidized and degraded. To evaluate whether the antioxidant activity suppresses the mitochondrial superoxide level in chondrocytes *in vitro* and *in vivo*, we selected a stable, permeable, and less toxic derivative of vitamin C, L-ascorbyl 2-phosphate 6-palmitate (APPS)^28^,^29^,^30^, to deliver and to attenuate the oxidative damage in chondrocytes. APPS treatment significantly suppressed the mitochondrial superoxide level in *Sod2* cKO chondrocytes at culture day 6 after APPS treatment for 24 h, confirming the protective role of APPS *in vitro* (Fig. 6a).

To determine whether APPS suppresses the mitochondrial superoxide generation in chondrocytes *in vivo*, APPS was injected into the joint cavity of the DMM side in *Sod2* cKO mice at 2 weeks after DMM surgery. *Sod2* cKO chondrocytes were isolated from the DMM-induced tibial plateau cartilage after an intra-articular injection of 1% APPS for 24 h. Mitochondrial superoxide in isolated chondrocytes was measured using flow cytometry with a DHE dye. The mitochondrial superoxide generation was significantly suppressed in *Sod2* cKO chondrocytes from the DMM side after the intra-articular injection of 1% APPS (Fig. 6b). In contrast, the vehicle injection failed to suppress the mitochondrial superoxide level in *Sod2* cKO chondrocytes from the DMM side after the intra-articular injection of phosphate buffered saline (PBS) (Fig. 6b). To evaluate the therapeutic effect of APPS in the cartilage, 1% APPS was intra-articularly injected once a week to the DMM side of knee joints in *Sod2* cKO mice from one week to eight weeks after surgery. The histological analysis of the knee joints clearly revealed that cartilage degeneration was significantly attenuated in the DMM side after the intra-articular injection of APPS compared with the PBS injection (Fig. 6c).

**Figure 7:**
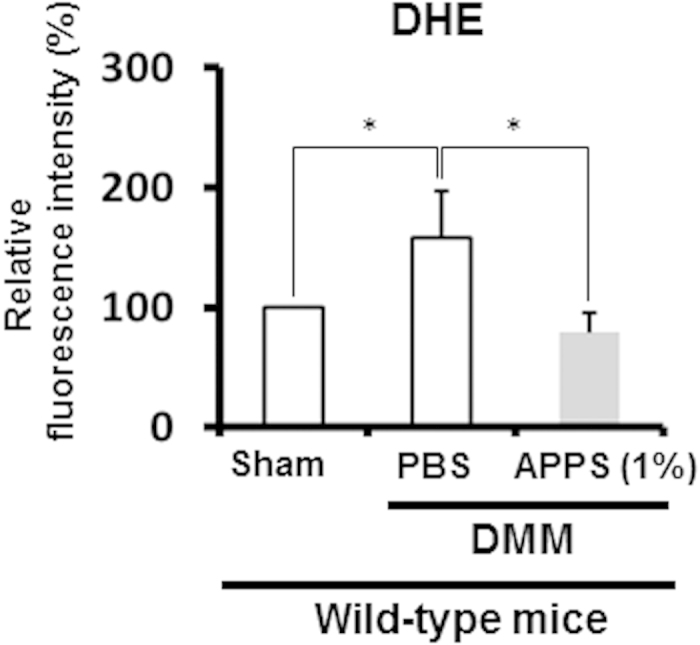
Antioxidant treatment effectively normalizes mitochondrial superoxide generation in wild-type articular chondrocytes *in vivo*. Superoxide generation in chondrocytes isolated from the tibial plateau of wild-type cartilage after the intra-articular injection of 1% APPS for 24 h was analyzed via flow cytometry. APPS or PBS was injected into the joint cavity on the DMM side in wild-type mice at 2 weeks after DMM surgery (*n* = 3, *P < 0.05 versus control, Student’s *t*-test).

Furthermore, to determine whether APPS suppresses mechanical loading-induced superoxide generation in chondrocytes of wild-type mice, 1% APPS was injected into the joint cavity of the DMM side in wild-type mice at 2 weeks after DMM surgery. Mitochondrial superoxide generation was significantly suppressed in the wild-type chondrocytes from the DMM side after the intra-articular injection of APPS (Fig. 7). These results demonstrated that APPS may be a useful antioxidant for suppressing mitochondrial superoxide generation in the cartilage chondrocytes.

## Discussion

### Mechanical overloading induced mitochondrial superoxide generation and *Sod2* downregulation in cartilage

We discovered that abnormal loading significantly elevated mitochondrial superoxide generation and downregulated the *Sod2* expression in wild-type chondrocytes from the DMM side compared with the sham side before the appearance of obvious cartilage degeneration *in vivo* (Fig. 1), suggesting a negative correlation between the mitochondrial superoxide leak and SOD2 decline. In a previous study, Wolff and Goodwin reported that the fluorescence intensity of DHE staining was increased in the bovine chondrocytes of osteochondral explants under mechanical stimuli^16^,^31^. Furthermore, Fermor *et al.* reported that intermittent high compression induced reactive oxygen and nitrogen species production in cartilage explant^32^,^33^. These data strongly suggested that mechanical overloading enhanced superoxide generation from the mitochondria in chondrocytes.

PQ treatment, which induces mitochondrial superoxide generation at complex I^26^, significantly suppressed the *Sod2* expression and mitochondrial function, resulting in the impairment of matrix biosynthesis in the chondrocytes (Fig. 2). Interestingly, Ji *et al.* have been revealed that a low dose treatment of H_2_O_2_ temporarily increased the SOD2 protein level in PC12 cells^34^. Conversely, a high dose treatment of H_2_O_2_ tended to decrease the *Sod2* mRNA and significantly decreased the SOD2 protein level via upregulation of miR-146a, which binds the region of *Sod2* 3’UTR, resulting in the post-transcriptional suppression of SOD2^34^. Recently, Jin *et al.* also reported that the miR-146a expression was increased in human chondrocytes after a mechanical pressure for 6 h^35^. Furthermore, Li *et al.* reported that abnormal loading significantly upregulated miR-146a expression in rats^36^. Moreover, Yamazaki *et al.* reported that miR-146a was intensively expressed in early stage of human OA cartilage^37^. These findings strongly suggested that abnormal loading triggered mitochondrial superoxide production and resulted in miR-146a upregulation accompanied with *Sod2* downregulation to continue and to accelerate mitochondrial oxidative damage in chondrocytes.

### Abnormal loading impaired mitochondrial superoxide balance in cartilage during aging

We have, for the first time, demonstrated that SOD2 loss in chondrocytes accelerated cartilage degeneration during aging and under an instability murine model (Figs 3 and 4). Three reports have previously described the selective downregulation of *SOD2* in knee OA cartilage. Aigner *et al.* revealed a decreased expression of *SOD2*, *SOD3*, and *GPX* via a microarray analysis using isolated chondrocytes from OA cartilage^20^. Ruiz-Romero *et al.* also reported that the SOD2 protein and gene expression in knee OA cartilage were significantly reduced compared with those from aged-matched normal knee cartilage from donors^21^. Scott *et al.* further reported that the *SOD2* gene expression in knee OA cartilage was significantly downregulated compared with normal femoral head cartilage^22^. In an animal study, the spontaneous OA chondrocytes from Hartley guinea pigs showed downregulation of the SOD2 protein before obvious cartilage degeneration^22^. These reports collectively indicated that a low level of SOD2 in chondrocytes negatively correlated with cartilage degeneration associated with chronic mechanical loading in human and animals.

The transient receptor potential vanilloid 4 (TRPV4) is a mechanical stress-sensing channel^38^,^39^. Bubolz *et al.* reported that a TRPV4 agonist increased the intracellular Ca^2+^ resulting in mitochondrial superoxide generation in human coronary arterioles^40^. O’Conor *et al.* also reported that TRPV4 loss accelerated cartilage degeneration in mice^41^. A previous *in vitro* study revealed that dynamic mechanical loading maintained the articular chondrocytes homeostasis via TRPV4 signaling^42^. These reports strongly suggested that TRPV4 signaling regulates mitochondrial superoxide generation in chondrocytes under mechanical loading.

Sirtuin 3 (SIRT3) is a member of the sirtuin family that promotes the lifespan of many organisms and is a stress-response deacetylase which is localized in the mitochondria in tissues^43^. Sundaresan *et al.* reported that SIRT3 transcriptionally and post-translationally regulated the SOD2 expression and activity in mitochondria via FoxO signaling to block cardiac hypertrophy^44^. Furthermore, the aging process gradually suppressed the SIRT3 expression in several tissues^45^, suggesting that SIRT3 directly regulates the mitochondrial function through SOD2 biosynthesis. These findings suggest that chronic mechanical stimuli can disturb the mitochondrial superoxide homeostasis via SIRT3-SOD2 axis in chondrocytes during aging.

### A vitamin C derivative effectively suppressed mitochondrial superoxide generation and delayed cartilage degeneration

Several reports indicated that antioxidants, such as NAC, and procyanidins, attenuated the OA development induced by instability in mice^46^,^47^. We have also previously reported that antioxidants, such as EUK-8, procyanidine, and vitamin C, ameliorated the age-related changes in the tissues of *Sod1* or *Sod2*-deficient mice^28^,^29^,^48^,^49^,^50^.

We selected a stable, permeable, and less toxic vitamin C derivative to suppress mitochondrial superoxide generation in chondrocytes *in vivo*. Indeed, Du *et al.* reported that APPS treatment can easily enter into cells and rapidly convert vitamin C, resulting in high concentration of intracellular vitamin C compared with the same dose treatment of vitamin C *in vitro*^30^. The APPS treatment in the joint cavity significantly normalized the mitochondrial superoxide level in chondrocytes, suggesting that APPS can convert vitamin C in cells, enter the mitochondria, and directly regulate mitochondrial superoxide generation *in vivo* (Figs 6 and 7). In addition, we revealed that the APPS treatment in the joint cavity significantly ameliorated cartilage degeneration in *Sod2* cKO mice (Fig. 6).

In 2002, Kraus and colleagues revealed that vitamin C promoted the synthesis of type II collagen and aggrecan in cartilage explants from guinea pigs^27^, proposing its beneficial effects for cartilage degeneration. However, Kraus *et al.* additionally reported that the oral intake of vitamin C increased the serum vitamin C level and worsened the natural-occurring cartilage degeneration in Dunkin-Hartley guinea pigs^51^. In contrast, Park *et al.* reported that the intra-articular injection of a nutritive mix solution including 5% vitamin C prevented the development of surgical-induced cartilage degeneration in a rabbit model^52^, suggesting that vitamin C effectively prevented cartilage degeneration via an intra-articular injection *in vivo*. In an epidemiological study, the serum vitamin C and E levels in OA patients were significantly higher than those of age-matched individuals^53^. Conversely, the vitamin C level in the joint fluid was significantly decreased in OA patients^54^, suggesting that the vitamin C level is reciprocally regulated between the serum and joint cavity in OA patients. These findings suggested that the oral intake of vitamin C may not sufficiently regulate the vitamin C level in the joint cavity in humans. In fact, Clark *et al.* reported that vitamin C solution showed a strong acidity leading to oxidative damage in cartilage explants *in vitro*^27^. Furthermore, vitamin C was found to induce apoptosis in human articular chondrocytes in a dose-dependent manner^55^. In fact, we also revealed that vitamin C treatment suppressed viability and downregulated *Col2a1* and *Acan* expression in wild-type chondrocytes (Supplementary Fig. S5b, c). In contrast, APPS treatment did not modify viability and upregulated *Col2a1* and *Acan* expression in wild-type chondrocytes (Supplementary Fig. S5b, c), indicating that the vitamin C-permeable derivative shows less side effect for chondrocyte viability. We therefore attempted to evaluate whether permeable non-acidic vitamin C can be delivered into the cartilage via an intra-articular injection and delay cartilage degeneration. Our findings revealed that a vitamin C-permeable derivative significantly suppressed the mitochondrial superoxide level and mitigated cartilage degeneration (Figs 6 and 7).

For future studies, the protocol of utilizing an intra-articular injection of a vitamin C derivative to ameliorate cartilage degeneration must be optimized. Our findings strongly suggested that permeable antioxidants with less adverse effects may be a promising therapy for OA development and progression.

## Methods

### Surgical-induced OA model (DMM model)

The mice were maintained and studied according to protocols approved by the Animal Care Committee of Chiba University. Surgical-induced OA model was induced as destabilization of the medial meniscus (DMM) by resecting the medial meniscotibial ligament (MMTL) as previously described^23^.

### Quantification of superoxide generation in chondrocytes isolated from the tibial plateau articular cartilage in mice

DMM was created in right knee joints, while a sham surgery was performed in the left knee joint of eight-week-old male mice. At 2 weeks after DMM surgery, tibial plateau cartilages from the DMM and sham sides were separately digested with Liberase in 6x digestion solution (6x digestion solution with the addition of 0.18 mg/mL Liberase TM Research Grade (Roche Applied Science, Mannheim, Germany), 100 unit/mL penicillin, and 0.1 mg/mL streptomycin in α-MEM (Life Technologies Corporation, Carlsbad, CA, USA)) for 2 h at 37 °C in a 20% O_2_ and 5% CO_2_ incubator. After incubation, culture medium was added to stop the digestion reaction. Digested cartilages were well suspended using a 1,000 μL tip and centrifuged for 5 min, 200 x *g* to obtain isolated chondrocytes. Pellets was re-suspended with phosphate buffered saline (PBS) and then centrifuged for 5 min, 200 x *g.* The isolated chondrocytes were stained for 30 min at 37 °C with 10 μM dihydroethidium (DHE, Life Technologies Corporation, Gaithersburg, MD, USA) or 5 μM MitoSOX (Life Technologies Corporation) to detect intracellular or mitochondrial superoxide, respectively^56^. Stained chondrocytes was then washed with PBS by centrifugation for 5 min, 200 x *g*, re-suspended in PBS, and then the superoxide level was quantified using a BD FACS Canto II flow cytometer (Becton Dickinson & Company, Franklin Lakes, NJ, USA).

### Isolation and culture of primary murine articular chondrocytes

Primary chondrocytes were prepared from femoral condyles and tibia plateaus of 6-day-old pups as previously described with some modifications^57^,^58^. The mice were sacrificed under general anesthesia. After washing with 70% ethanol, the skin and soft tissues of the lower limbs were removed using tweezers. The articular cartilage, including femoral condyles and tibia plateaus, were isolated using scissors and washed with sterile PBS (Step 1). To remove the soft tissues, these pieces were transferred to 6x digestion solution (250 μL/well) in a 48-well plate (BD Falcon™), and then incubated for 45 min at 37 °C in a 20% O_2_ and 5% CO_2_ incubator (Step 2). To remove the residual soft tissues, cartilage pieces were agitated using a sterile plastic 1,000 μL tip (Step 3). Steps 1 to 3 were repeated once. To isolate the chondrocytes from the cartilage, cartilage pieces were transferred to 1x digestion solution (consisting of α-MEM supplemented with 0.03 mg/mL Liberase TM Research Grade, 100 unit/mL penicillin, and 0.1 mg/mL streptomycin) (250 μL/well) in a 48-well plate, and then incubated overnight at 37 °C in a 20% O_2_ and 5% CO_2_ incubator. After incubation, 750 μL serum-free medium (consisting of α-MEM supplemented with 100 unit/mL penicillin, and 0.1 mg/mL streptomycin) was added to the digestion solution containing the residual chondrocytes, and was well suspended using a 1,000 μL tip to dissociate the cells. Then, the cell suspension was passed through a 48 μm cell strainer into a fresh 15 mL polypropylene tube and centrifuged for 5 min, 200 x *g* to obtain isolated chondrocytes. The pellets were washed with 5 mL PBS, centrifuged for 5 min, 200 x *g*, and the pellet was re-suspended with culture medium (consisting of α-MEM supplemented with 10% fetal bovine serum (FBS, HyClone, Thermo Scientific, USA), 100 unit/mL penicillin, and 0.1 mg/mL streptomycin). Approximately 3.0 − 6.0 × 10^5^ articular chondrocytes were isolated at per mouse. Primary chondrocytes were seeded at a density of 8,000 cells/cm^2^ in plastic dishes.

### Paraquat treatment

Methyl viologen dichloride hydrate (paraquat, Sigma-Aldrich, St. Louis, MO, USA) was dissolved in PBS to create a stock solution of 10 mM and was used at a final concentration of 1 mM. At culture day 5, primary articular chondrocytes were treated with 1 mM paraquat (PQ) for 24 h.

### Measurement of mitochondrial membrane potential

Primary articular chondrocytes were stained with JC-1 dye (Life Technologies Corporation) as previously described^56^ with some modifications. Cultured chondrocytes were washed with PBS, trypsinized for 5 min at 37 °C under 20% O_2_ and centrifuged for 5 min, 200 x *g*. The pellet was then re-suspended in culture medium, and cells were stained for 15 min at 37 °C with 10 μM JC-1 in FBS-free medium. After staining, the cells were washed and re-suspended in PBS, and then the mitochondrial membrane potential was measured using a BD FACS Canto II flow cytometer.

### Mitochondrial morphology in primary articular chondrocytes

At culture day 6, primary articular chondrocytes were fixed in 2.5% glutaraldehyde in PBS for 60 min at room temperature. The mitochondrial morphology of primary articular chondrocytes was evaluated using a Hitachi HT7700 electron microscope (HITACHI high-tech, Tokyo, Japan).

### Oxygen consumption rate (OCR) in primary articular chondrocytes

OCR values in primary articular chondrocytes were measured using the Seahorse XF-96 extracellular flux analyzer (Seahorse Biosciences, MA, USA) as previously described^59^. Primary chondrocytes were plated in Seahorse XF96 cell culture microplates at a density of 1 × 10^4^ cells/well and cultured for five days. At culture day 6, cells were washed twice with serum-free DMEM, and then incubated in serum free DMEM supplemented with 25 mM glucose and 1 mM pyruvic acid. The respirometer was then calibrated according to the manufacturer’s instructions, and cells were treated with oligomycin (ATP synthase inhibitor), FCCP, rotenone (mitochondrial complex I inhibitor), and antimycin A (mitochondrial complex III inhibitor). OCR was measured after every treatment.

### Skeletal preparation

Whole skeletons of *Sod2* cKO and wild-type littermates control mice on postnatal day 6 were stained with Alcian blue and Alizarin red as previously described^60^.

### Generation of chondrocytes-specific *Sod2* conditional knockout mice

*Col2a1*-Cre/+ transgenic mice on a C57BL6/J background were obtained from The Jackson Laboratory (Cre under the control of the collagen type II promoter JAX#003554, Bar Harbor, ME, USA)^61^. *Sod2*^*fl/fl*^ mice on a C57BL/6NCrSlc were generated as previously described^62^,^63^,^64^. These mice were crossbred to generate chondrocytes-specific *Sod2* conditional knockout mice (*Col2a1-Cre;Sod2*^*fl/fl*^). All genotyping was performed via genomic PCR. The primers used to identify the *Col2a1*-Cre/+ transgene allele are as follow: 5’-GTG AAA CAG CAT TGC TGT CAC TT-3’ and 5’-GCC ACG TTG TGA GTT GGA TAG TTG-3’, under the following PCR conditions: 1 cycle of 94 °C for 5 min and 35 cycles of 94 °C for 0.5 min, 58 °C for 0.5 min, and 72 °C for 1 min followed by 1 cycle of 72 °C for 5 min. *Sod2* flox alleles were detected as previously described^62^.

### Histological evaluation of spontaneous OA model

At 12 months of age, male *Sod2* cKO and wild-type littermates were histologically evaluated in the knee joints under the spontaneous OA model as previously described with some modifications^65^. Entire knee joints were fixed in 4% paraformaldehyde phosphate buffer solution (Wako Pure Chemical Industries, Ltd, Osaka, Japan) for 48 h at 4 °C, decalcified for 2 weeks with 20% EDTA at 4 °C on a shaker, and embedded in paraffin wax. Each embedded knee joint was cut into 5 μm slices at the medial sagittal plane. The cut specimen was then stained with Fast Green FCF, pure (Waldeck GmbH & Co. KG, Germany) for 5 min and safranin O (Sigma-Aldrich) for 30 min as previously described with some modifications^66^. The histological OA grade was evaluated using the OARSI cartilage OA histopathology grading system (score 0–6)^65^.

### Locomotive activity

Locomotive activity was monitored using implanted transmitting devices as previously described^67^. Scores were obtained as counts per one hour, and the 24 h profile of daily activity was obtained by averaging seven days of continuous data.

### Generation of tamoxifen-inducible *Sod2* knockout mice

*Rosa26-Cre*^*ERT2*^ transgenic mice on a C57BL6/J background in which the Cre recombinase is fused to a mutated ligand-binding domain of the human estrogen receptor driven by the *Rosa26* promoter were obtained from The Jackson Laboratory (JAX#004847, Bar Harbor, ME, USA)^68^. *Sod2*^*fl/fl*^ mice on a C57BL/6NCrSlc were generated as previously described^62^,^63^. These mice were crossbred to generate tamoxifen-inducible *Sod2*-deficient mice (*Rosa26-Cre*^*ERT2*^*;Sod2*^*fl/fl*^). All genotyping was performed via genomic PCR. The primers used to identify the *Rosa26–Cre*^*ERT2*^ transgene allele were as follows: 5’-GCG GTC TGG CAG TAA AAA CTA TC-3 and 5’-GTG AAA CAG CAT TGC TGT CAC TT-3’, under the following PCR conditions: 1 cycle of 94 °C for 3 min and 35 cycles of 94 °C for 0.5 min, 63 °C for 1 min, and 72 °C for 1 min followed by 1 cycle of 72 °C for 2 min. *Sod2* flox and *Sod2* deletion alleles were detected as previously described^62^.

### Cell culture of primary articular chondrocytes with tamoxifen treatment

4-Hydroxy-tamoxifen (4-OHT, Calbiochem, Darmstadt, Germany) was dissolved in DMSO (Sigma-Aldrich) at a stock solution of 1 mM and used at a final concentration of 1 μM. We isolated articular chondrocytes from *Sod2*^*fl/fl*^ and *Rosa26-Cre*^*ERT*^*;Sod2*^*fl/fl*^ mice. Primary chondrocytes were seeded at a density of 8,000 cells/cm^2^ in plastic dishes. After 24 h, we treated the cells with 1 μM 4-OHT or DMSO, and replaced the cultures with freshly prepared medium once every other day.

### Histological evaluation of the DMM model in *Sod2* cKO mice

To evaluate the knee joints histologically, DMM was created in the right knee joints of male *Sod2* cKO and wild-type littermates at eight weeks of age, while sham surgeries (resecting the joint capsule only) were performed in the left knee joints of male *Sod2* cKO and wild-type littermates at eight weeks of age. Mice were sacrificed at eight weeks of age after the DMM surgery, and then entire knee joints were fixed in 4% paraformaldehyde plus 0.1 M PBS solution for 48 h at 4 °C, decalcified for 2 weeks with 20% EDTA at 4 °C on a shaker, and embedded in paraffin wax. Each specimen from the surgical-induced OA model was cut into 5 μm slices at the coronal plane at six slice intervals per knee joint. The specimens were stained with Fast Green for 5 min and safranin O for 30 min. The histological OA grade was evaluated using the OARSI cartilage OA histopathology grading system^65^. Four quadrants of the joint, including the medial femoral condyle (MFC), lateral femoral condyle (LFC), medial tibial plateau (MTP), and lateral tibial plateau (LTP), were scored and the quadrant score of the six coronal sections was summed. Additionally, MFC and MTP were each scored individually. OA grading was assessed by a single observer who was blinded to the study.

### Superoxide generation in primary articular chondrocytes

Cultured chondrocytes were washed with PBS and trypsinized for 5 min at 37 °C under 20% O_2_ condition. FBS-free medium was added and trypsinized cells were collected and centrifuged for 5 min, 200 x *g* to isolate chondrocytes. The cells were stained and incubated for 30 min at 37 °C with 10 μM DHE in culture medium. Then, the cell suspension was washed with PBS, and staining was visualized using a BD FACS Canto II flow cytometer.

Tamoxifen-inducible *Sod2* deficient chondrocytes were stained for 30 min at 37 °C with 10 μM DHE. After staining, the culture dishes were washed twice using PBS and then re-suspended in PBS. The fluorescence image was evaluated by a microfluorescence system (AF6500; Leica Microsystems, Wetzlar, Germany) and captured using the AF6500 software program.

### Western blotting

The chondrocytes were lysed in an NP-40 lysis buffer previously described^56^. The samples were placed at 4 °C for 20 min, vortexed, and centrifuged at 20,000 x g for 15 min. The supernatant was assayed for protein concentration with a DC Protein Assay Kit (BioRad, Hercules, CA), and 5 μg of each sample were loaded onto a 10% SDS-polyacrylamide gel. Antibodies against SOD1 (ADI-SOD-100-F, Enzo Life Sciences), SOD2 (ADI-SOD-200-F, Enzo Life Sciences), catalase (#8841S, Cell Signaling Technology), GPX1 (#3206S, Cell Signaling Technology), and actin (A2066, Sigma-Aldrich, St. Louis, MO, USA) were used.

### Quantification of Alcian blue staining

The proteoglycan production in chondrocytes was evaluated using Alcian blue (Muto Pure Chemicals, Tokyo, Japan) staining as previously described^58^. Stained chondrocytes were captured by a flathead scanner, and then images were quantified via QWin image analysis software (Leica, Wetzlar, Germany).

### Quantitative real-time PCR

Total RNA was extracted from chondrocytes using the RNeasy Mini Kit according to the manufacturer’s instructions (Qiagen Inc., Valencia, CA, USA). Complementary DNA (cDNA) was synthesized from 1 μg of total RNA using the ReverTra Ace qPCR RT Kit (TOYOBO, Japan). Total cDNA (100 ng) was used as a template for the real-time RT-PCR analyses. cDNA was quantified using an ABI Prism 7500 sequence-detection system with SYBR Green PCR Master Mix (Applied BioSystems, Foster City, CA, USA) according to the manufacturer’s instructions. The detector was programmed with the following PCR conditions: 40 cycles of 15 s of denaturation at 95 °C and 1 min of amplification at 60 °C. All reactions were performed in monoplicate and *Gapdh* was used as an endogenous control. The relative differences in the PCR results were calculated using the comparative cycle threshold method. The primer sets used in this study are summarized in Table 1 (supplementary information).

### Treatment with a vitamin C derivative

L-ascorbyl 2-phosphate 6-palmitate (APPS, Lot No. L-101203, Showa Denko K. K., Tokyo, Japan) was dissolved in PBS at a stock solution of 10 mM and used at a final concentration of 1 μM *in vitro*. Twenty microliters of APPS at a final concentration of 1% (w/v) was injected into knee joints using a 30 G needle as previously described *in vivo*^69^. Superoxide generation in primary chondrocytes was measured at culture day 6 after 10 μM APPS treatment for 24 h *in vitro*. A flow cytometoric analysis was conducted to measure superoxide generation in chondrocytes isolated from the tibial plateau of *Sod2* cKO cartilage after the intra-articular injection of 1% APPS or PBS for 24 h *in vivo*. 1% (w/v) APPS was intra-articularly injected once a week to the DMM side of knee joints in mice for eight weeks after surgery. The medial compartment in knee joints from the DMM side was histologically evaluated at eight weeks after DMM surgery using the OARSI score.

## Additional Information

**How to cite this article**: Koike, M. *et al.* Mechanical overloading causes mitochondrial superoxide and SOD2 imbalance in chondrocytes resulting in cartilage degeneration. *Sci. Rep.* **5**, 11722; doi: 10.1038/srep11722 (2015).

## Supplementary Material

Supplementary Information
