# Supplementary material for: Mechanical overloading causes mitochondrial superoxide and SOD2 imbalance in chondrocytes resulting in cartilage degeneration
Source: Sci Rep. 2015 Jun 25;5:11722. doi: 10.1038/srep11722 (PMC4480010; doi:10.1038/srep11722)
Supplement: Supplementary Information [file srep11722-s1.pdf]

Supplementary Online Material for

Mechanical overloading causes mitochondrial superoxide and SOD2 imbalance in chondrocytes resulting in cartilage degeneration

Masato Koike, Hidetoshi Nojiri, Yusuke Ozawa, Kenji Watanabe, Yuta Muramatsu, Haruka Kaneko, Daichi Morikawa, Keiji Kobayashi, Yoshitomo Saita, Takahisa Sasho, Takuji Shirasawa, Koutaro Yokote, Kazuo Kaneko, Takahiko Shimizu

Correspondence should be addressed to Takahiko Shimizu (shimizut@chiba-u.jp)

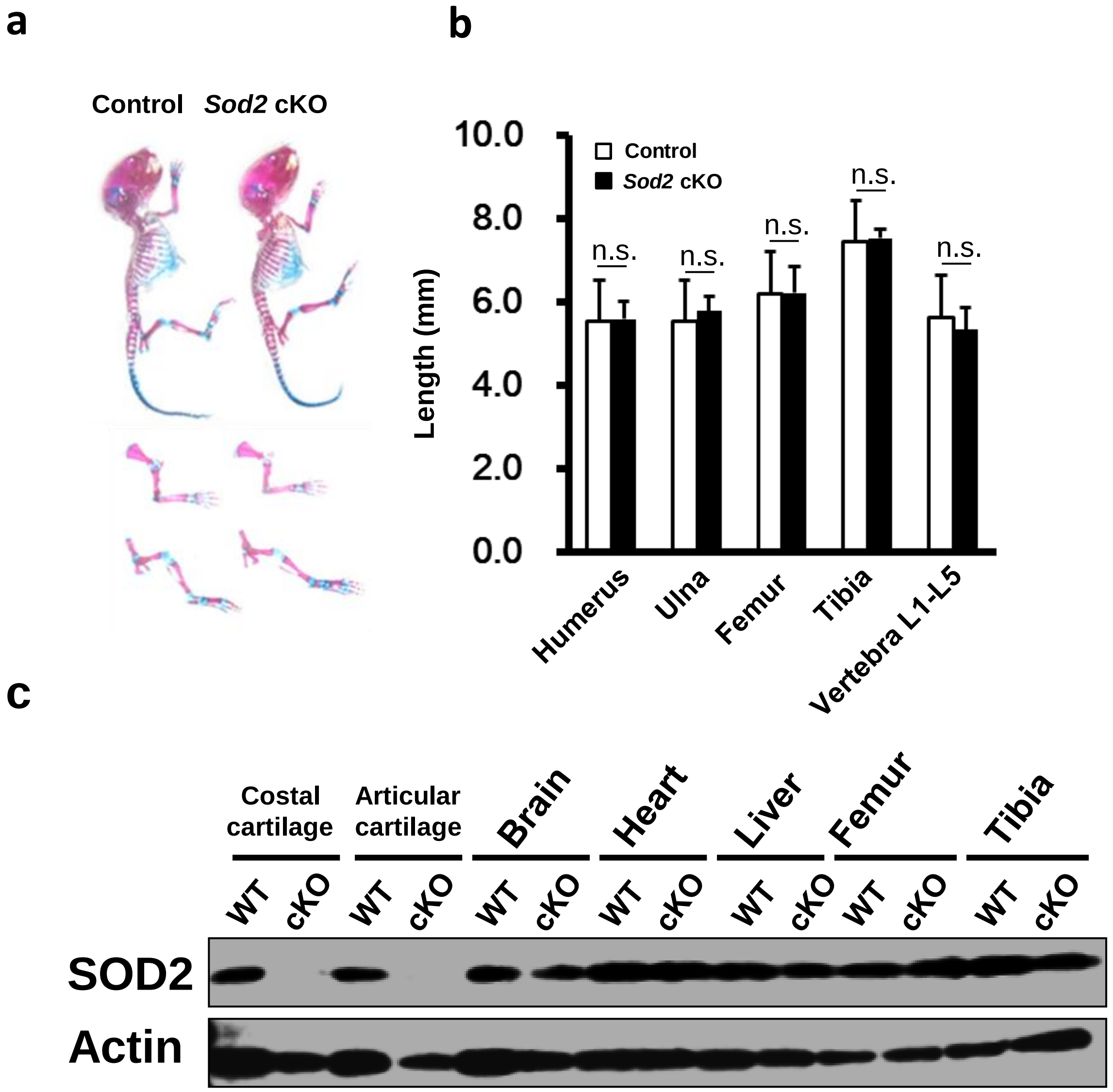

Supplementary Figure S1.

Generation of chondrocytes-specific *Sod2* knockout (*Sod2* cKO) mice. (**a**) Skeletal preparation of chondrocytes-specific *Sod2* cKO mice on postnatal day 6. (**b**) Quantification of the body length of long bones and vertebra (first to fifth lumbar spines) of control and *Sod2* cKO littermate on postnatal day 6 (n =4 - 6, n.s.: not significant versus control, Student's t-test). (**c**) SOD2 protein level in various tissues. WT: Control, cKO: *Sod2* cKO.

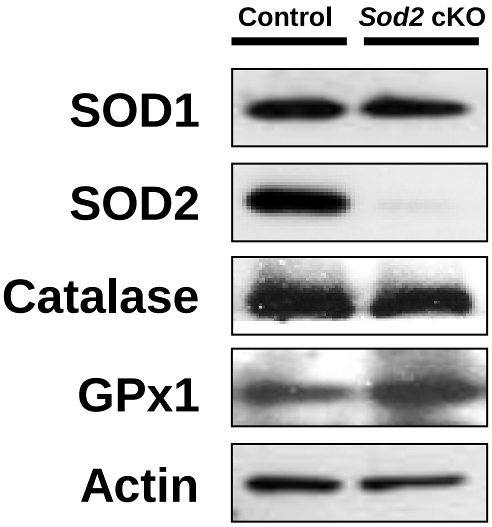

Supplementary Figure S2.
Protein levels of antioxidant enzymes in *Sod2* cKO chondrocytes. Primary articular chondrocytes were isolated from knee joints of neonatal control and *Sod2* cKO mice at culture day 6. Protein levels were evaluated by the western blot analysis using specific antibodies.

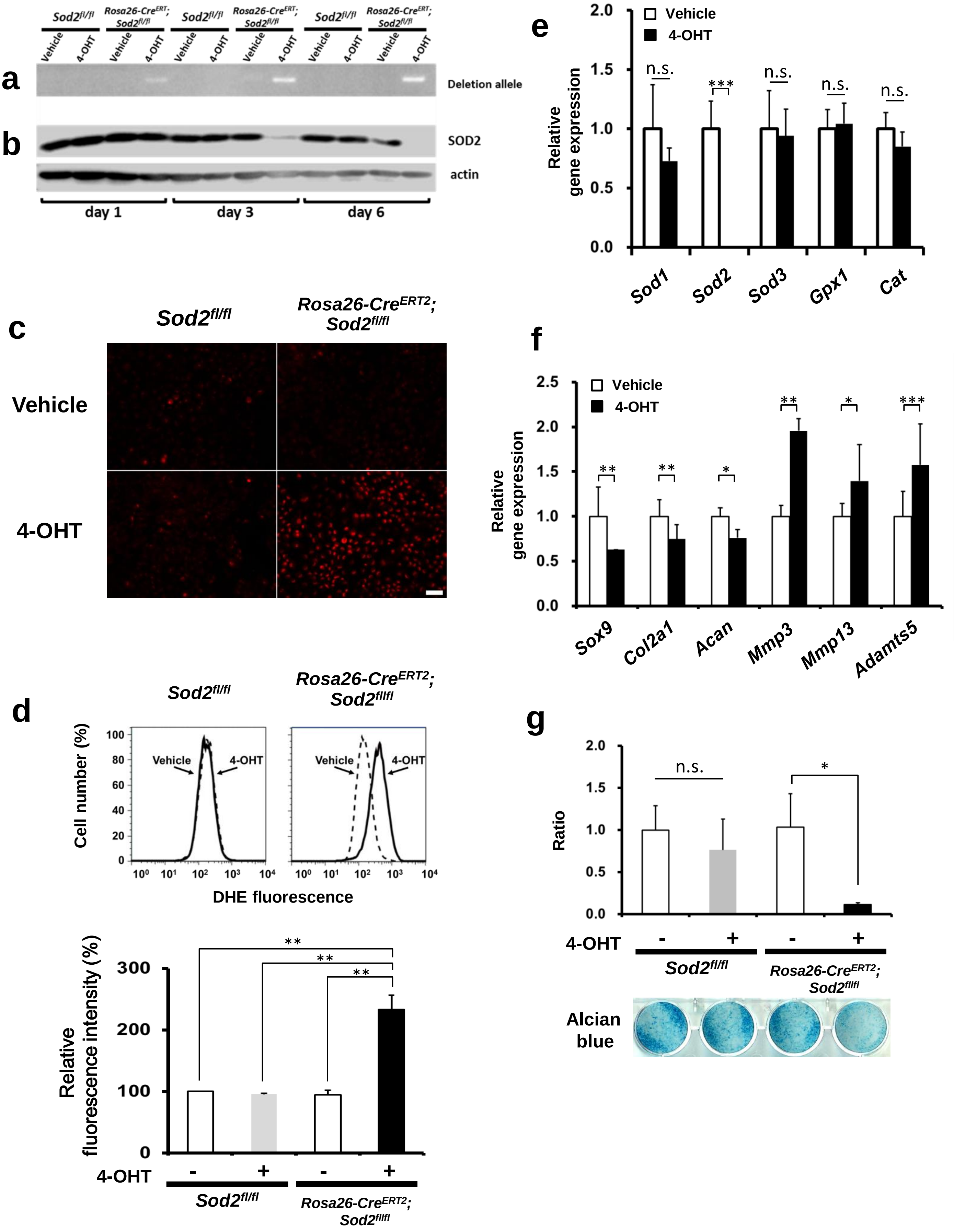

Koike *et al.,* Supplementary Figure S3

Supplementary Figure S3.

Tamoxifen-inducible *Sod2* deficiency in chondrocytes significantly induced superoxide overproduction and dysfunction resulting in impaired extracellular matrix homeostasis. (**a**) Genomic PCR analysis of cultured chondrocytes isolated from the knee joints of *Sod2*$^{fl/fl}$ and *Rosa26-Cre*^{ERT2}*;Sod2*$^{fl/fl}$ neonatal mice. 4-OH tamoxifen (4-OHT) and DMSO as a vehicle were added to culture medium to delete the *Sod2* gene. 4-OHT treatment effectively ablated the *Sod2* gene in *Rosa26-Cre*^{ERT2}*;Sod2*$^{fl/fl}$ chondrocytes in a time-dependent manner. (**b**) The SOD2 protein level in cultured primary articular chondrocytes at culture day 1, 3, and 6 after 4-OHT treatment. (**c**) The fluorescence microscopic analysis of tamoxifen-inducible *Sod2*-deficient chondrocytes by DHE staining at culture day 6. Scale bars represent 50 µm. (**d**) The flow cytometric analysis of cultured tamoxifen-inducible *Sod2*-deficient chondrocytes by DHE staining at culture day 6. The upper panels demonstrate the histogram and the lower panels indicate the quantification of superoxide generation. Data shown are frome three independent experiments. (**e, f**) Gene expression of antioxidant enzymes (**e**) and OA-related genes (**f**) in tamoxifen-inducible *Sod2*-deficient chondrocytes at culture day 6. Error bars show the mean ± s.d. of five mice per genes (*$P < 0.05$, **$P < 0.01$, ***$P < 0.001$ versus vehicle, n.s.: not significant, Student's *t*-test). (**h**) Proteoglycan levels in tamoxifen-inducible *Sod2*-deficient chondrocytes at culture day 21 using Alcian blue staining (*$P < 0.05$ versus control, n.s.: not significant, the Tukey test). Quantification of Alcian blue staining was achieved by QWin software. Data were analyzed using a Student's *t*-test (**a**), (**b**), (**c**), (**e**), and (**f**). The data of (**d**) and (**g**) were analyzed using the Tukey test and the mean comparison showed to be statistically significant (**$P < 0.01$), n.s.: not significant.

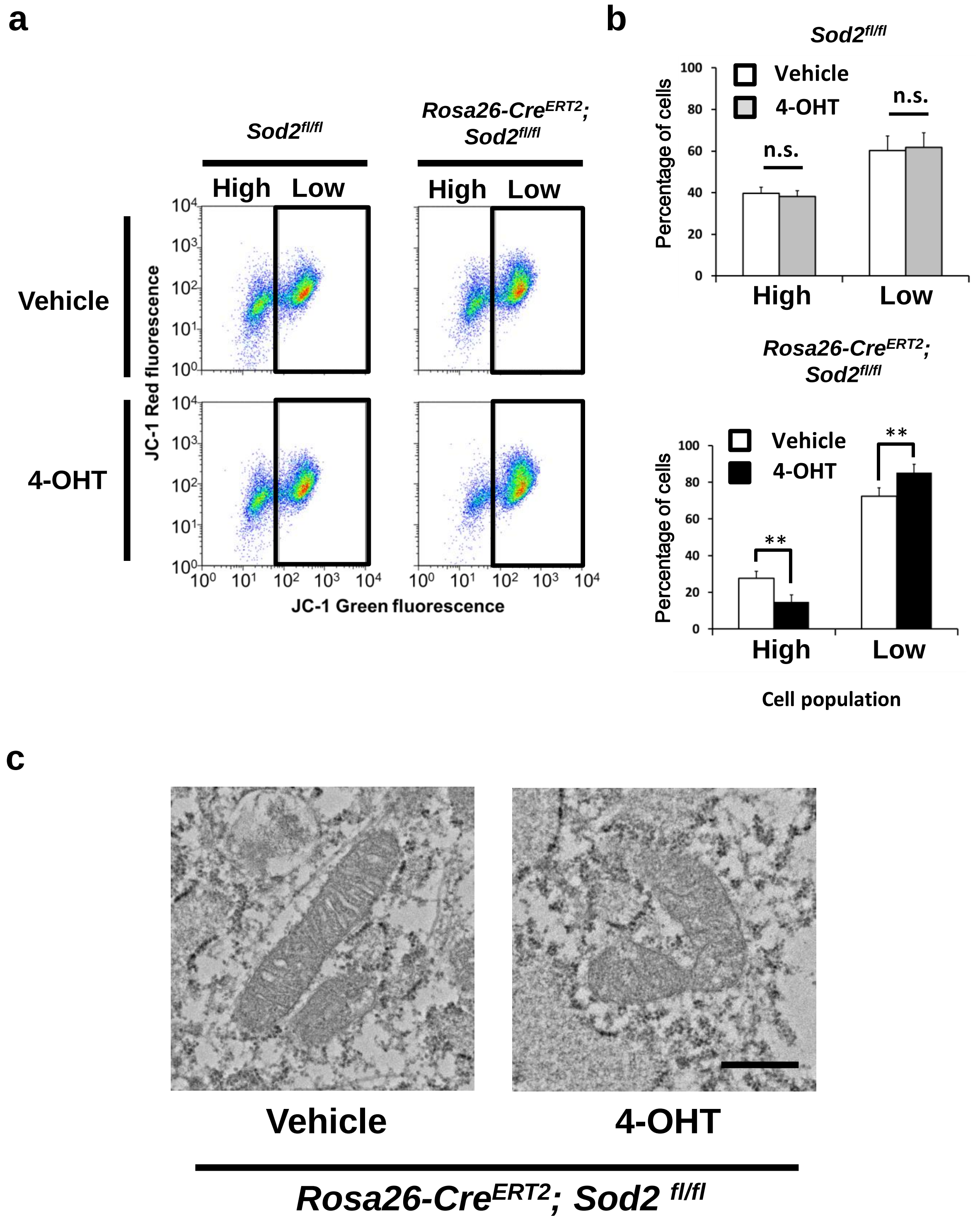

Supplementary Figure S4.

Mitochondrial superoxide significantly increases mitochondrial depolarization in tamoxifen-inducible *Sod2*-deficient chondrocytes. (**a**) Tamoxifen-inducible *Sod2*-deficient chondrocytes with low red and high green fluorescence (cells with mitochondrial depolarization) at culture day 6. High: the region of cells with normal ΔΨm, Low: the region of cells with mitochondrial depolarization. (**b**) The relative percentage of mitochondria with low mitochondrial membrane potential (ΔΨm) in chondrocytes at culture day 6. Values are the mean ± s.d. (n = 4, n.s., not significant versus control mice, **P < 0.01 versus vehicle, n.s.: not significant, Student's t-test). (**c**) Mitochondrial morphology in chondrocytes at culture day 6. Tamoxifen-inducible *Sod2*-deficient chondrocytes exhibited significant fewer cristae in the mitochondria. The left panel indicates the vehicle (*Rosa26-Cre^{ERT2};Sod2$^{fl/fl}$* vehicle) and the right panel indicates *Sod2*-deficient chondrocytes (*Rosa26-Cre^{ERT2};Sod2$^{fl/fl}$* 4-OHT). Scale bar represents 500 nm.

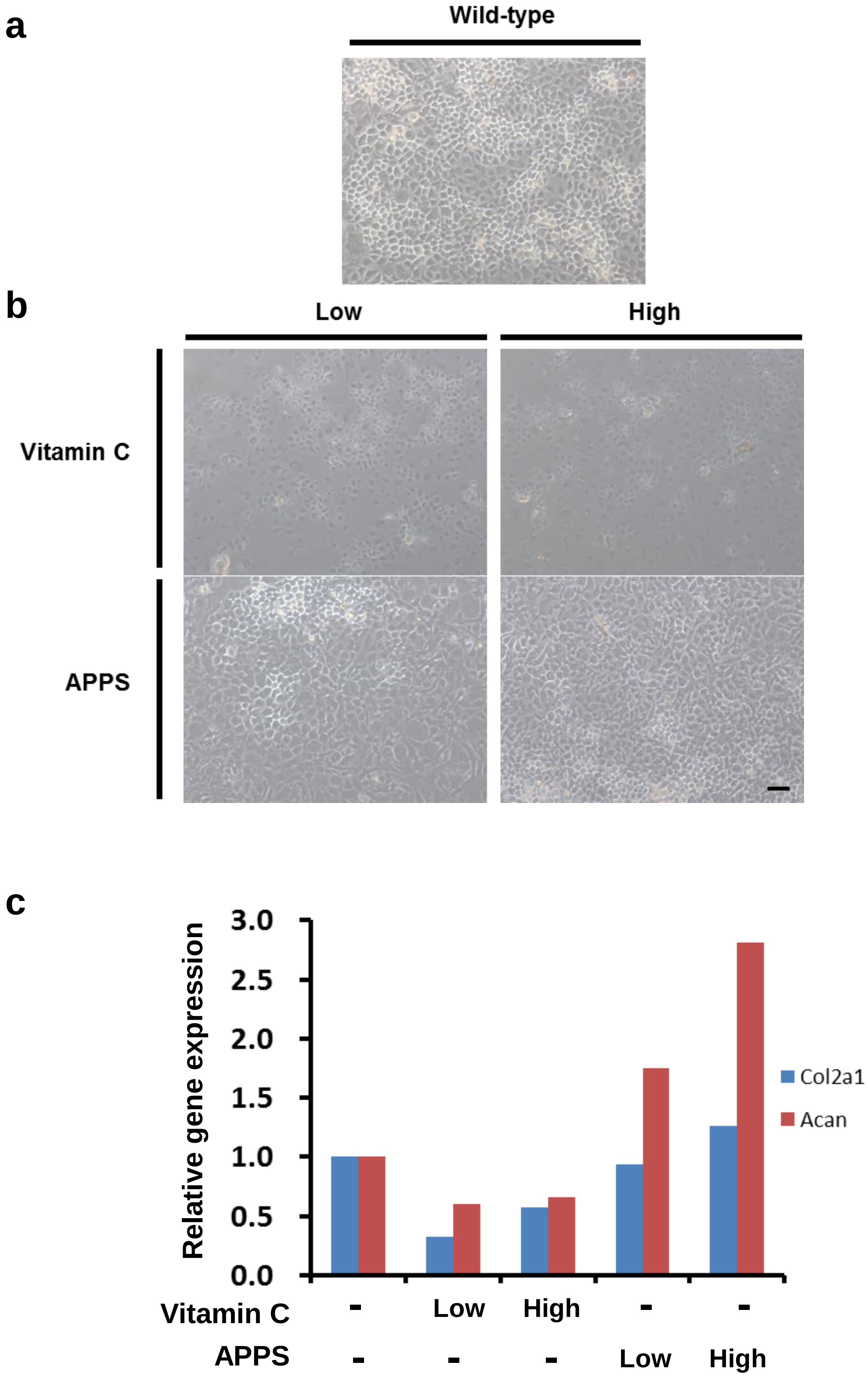

Supplementary Figure S5.

Chondrocyte viability after vitamin C or vitamin C derivative treatment. (**a**) Primary articular chondrocytes from B6 mice at culture day 6. (**b**) Cell viability of primary articular chondrocytes after vitamin C (L-Ascorbic acid, Sigma Aldrich) or APPS (vitamin C derivative) treatment for 24 h at culture day 6. Low: 31.25 µM, High: 125 µM. Upper panels indicate wild-type primary chondrocytes after vitamin C treatment. Lower panels indicate wild-type primary chondrocytes after vitamin C derivative (APPS) treatment. Scale bars represent 100 µm. (**c**) Gene expression of ECM related gene in wild-type primary chondrocytes after vitamin C or APPS treatment for 24 h at culture day 6. Low: 31.25 µM, High: 125 µM.

Supplementary Fig S1

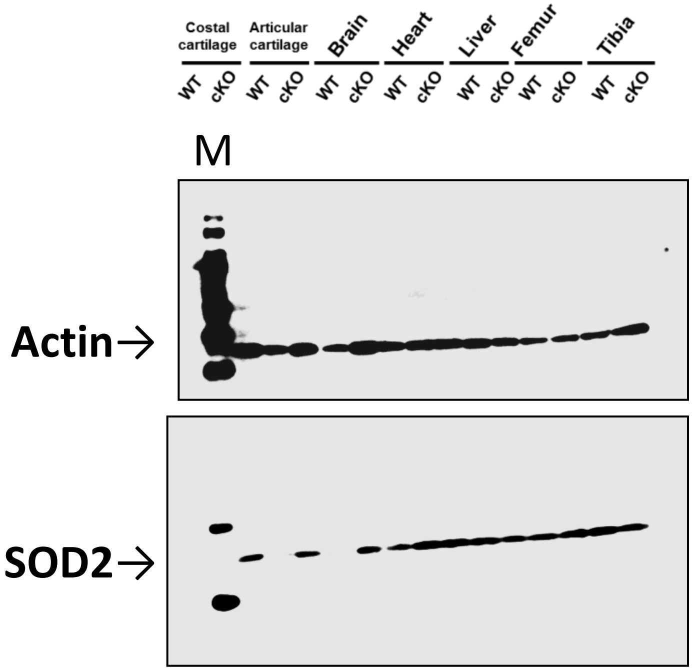

Supplementary Fig S3

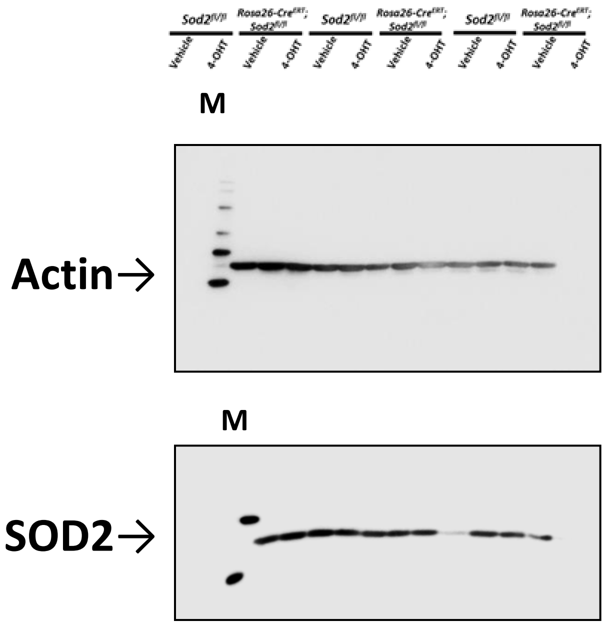

Supplementary Fig S2

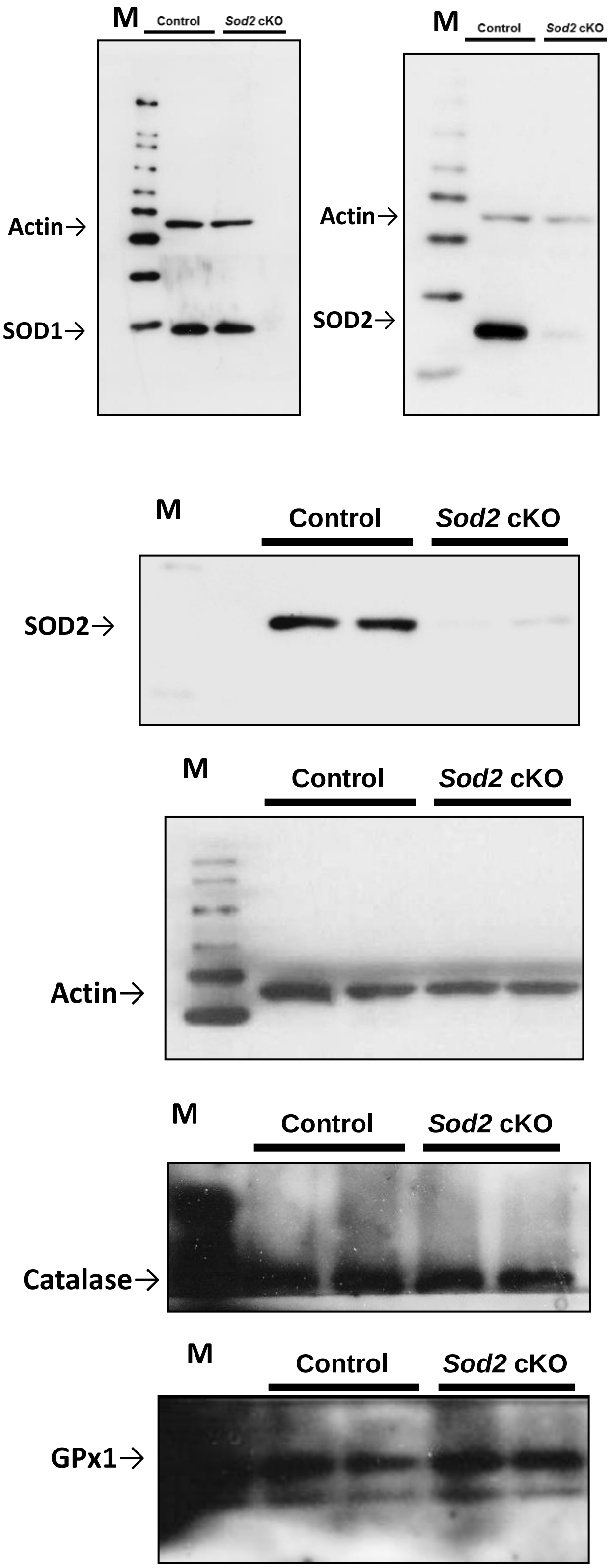

Koike *et al.* Supplementary Figure S6

Supplementary Table 1. Primers for real-time PCR

Gene		Primer Sequence
Sox9	Forward	GGA CCA CAC ATG CCT CTG CAA
	Reverse	TCT CCA GCC ACA GCA GTG AGT AA
Col2a1	Forward	GGC AAC AGC AGG TTC ACA TA
	Reverse	ATG GGT GCG ATG TCA ATA AT
Acan	Forward	CAG AGT TAG TGG AGG GTG TGA
	Reverse	AGA CCC TGG GAA GTT TGT
Mmp3	Forward	TGT GTG CTC ATC CTA CCC ATT GC
	Reverse	CCC TGT CAT CTC CAA CCC GAG GA
Mmp13	Forward	AGG CCT TCA GAA AAG CCT TC
	Reverse	TCC TTG GAG TGA TCC AGA CC
Adamts5	Forward	CCT GGC GGT GGT GAA GGT GG
	Reverse	TGC CCA CAT AAA TCC TCT CGG GTG A
Sod1	Forward	GCG GTG AAC CAG TTG TGT TGT C
	Reverse	CAG TCA CAT TGC CCA GGT CTC C
Sod2	Forward	ATG TTA CAA CTC AGG TCG CTC TTC
	Reverse	TGA TAG CCT CCA GCA ACT CTC C
Sod3	Forward	CTC TTG GGA GAG CCT GAC A
	Reverse	GCC AGT AGC AAG CCG TAG AA
Gpx1	Forward	GTC CAC CGT GTA TGC CTT CT
	Reverse	TCT GCA GAT CGT TCA TCT CG
Cat	Forward	ACA TGG TCT GGG ACT TCT GG
	Reverse	CAA GTT TTT GAT GCC CTG GT
Gapdh	Forward	AGA AGG TGG TGA AGC AGG CAT C
	Reverse	CGA AGG TGG AAG AGT GGG AGT TG
Rela	Forward	TGC CCA GAC CGC AGT ATC
	Reverse	GGA TTC GCT GGC TAA TGG
Ptgs2	Forward	CTG CTG CCC GAC ACC TTC AAC A
	Reverse	CAT TTC TTC CCC CAG CAA CCC GG
